# Draft Genome Sequence of *Pasteurella multocida* A:3 Strain 671/90

**DOI:** 10.1128/genomeA.00803-13

**Published:** 2013-10-03

**Authors:** Frederick A. Lainson, Mark P. Dagleish, Michael C. Fontaine, Colin Bayne, J. Christopher Hodgson

**Affiliations:** Moredun Research Institute, Edinburgh, United Kingdom

## Abstract

*Pasteurella multocida* serogroup A is commonly isolated from nasal swabs of clinically healthy calves and also from diseased lung tissue in bovine pneumonia. Here, we report the draft genome sequence of the virulent strain *P. multocida* 671/90, which has been characterized previously in experimental infections of calves and mice.

## GENOME ANNOUNCEMENT

The Gram-negative, facultatively anaerobic bacterium *Pasteurella multocida* is commonly found in the nasopharynx of clinically healthy calves. However, it is also a principal bacterial respiratory pathogen in farm cattle worldwide, causing morbidity and mortality with consequent social and economic costs. Hotchkiss et al. (1) reported a high prevalence of *P. multocida* in randomly sampled beef suckler and dairy calves in Scotland. *P. multocida* strain 671/90 is an A:3 serotype isolated from infected calf lung tissue following experimental infection with an isolate obtained after challenging calves intrabronchially with a mixture of 5 field isolates from cases of bovine pneumonia in England and Scotland. The A:3 serotype is most commonly associated with severe suppurative bronchopneumonia in calves, and strain 671/90 has been shown to be pathogenic following experimental infection in calves. Dagleish et al. (2) described the clinical responses and lung and lymph node pathologies in a group of calves over a period of 10 days during the course of progressive disease, showing that the pathological lesions occurred in lung tissue early in infection, before clinical signs developed. Taken together with the limited effectiveness of antimicrobials against this pathogen, this finding indicates that an effective vaccine would be the preferred means of mitigating the pathological consequences of natural infection.

Strain 671/90 was characterized in a multilocus sequence typing (MLST) survey of representative *P. multocida* strains from the United Kingdom and worldwide (3). While it had a unique profile and was assigned to a unique sequence type (ST), ST-89, eBURST analysis showed it to lie within the range of bovine respiratory isolates, although outside the clonal complex 13 (CC13) that defined the majority of these isolates.

Sequencing was carried out using combined Illumina PE and 454 platforms and assembled using Velvet version 1.0 (4) and Newbler, which were then coassembled using Minimus (5). This produced 26 contigs ranging in size from 124 to 578,571 bp, with a cumulative size of 2,246,063 bp and an N_50_ of 311,791 bp. Annotation was carried out using the NCBI Prokaryotic Genome Automatic Annotation Pipeline (http://www.ncbi.nlm.nih.gov/genome/annotation_prok/). This showed the G+C content to be 40.25% and predicted 2,115 genes, of which 2,064 were protein coding, 5 encoded rRNAs, and 46 encoded tRNA sequences.

Alignment of the contigs of strain 671/90 with the previously sequenced reference strain *P. multocida* PM70 (6) (accession no. NC_002663) using Nucmer (7) indicated extensive homology: 95.98% of the 671/90 genome mapped with an identity of ≥98%. Alignments were also visualized using Mapview (7) to show extensive regions of colinearity between these sequences.

### Nucleotide sequence accession numbers.

The draft genome sequences for *P. multocida* strain 671/90 have been deposited at NCBI as BioProject no. PRJNA188485 and are available from DDBJ/EMBL/GenBank under the accession no. ARWR00000000. The version described here is ARWR00000000.1.
